# The impact of exercise on improving sarcopenia in patients with liver cirrhosis: a systematic review and meta-analysis

**DOI:** 10.3389/fmed.2026.1871179

**Published:** 2026-07-29

**Authors:** Niezi Wang, Wei Sun, Yang Chen, Shuyuan Cong, Yixin Zhan

**Affiliations:** School of Physical Education, Xi’an Jiaotong University, Xi’an, China

**Keywords:** exercise, liver cirrhosis, meta-analysis, sarcopenia, systematic review

## Abstract

**Background:**

Sarcopenia and frailty are common issues faced by individuals suffering from liver cirrhosis. These ailments greatly diminish life quality and increase the likelihood of death. Engaging in physical activity is a popular method to improve sarcopenia. Consequently, this systematic review and meta-analysis seeks to assess the effectiveness of exercise programs for addressing sarcopenia in those with liver cirrhosis.

**Purpose:**

This study evaluated the efficacy of exercise interventions on sarcopenia in patients with liver cirrhosis through a meta-analysis of randomized controlled trials (RCTs).

**Methods:**

A comprehensive literature search was conducted across five databases: PubMed, Embase, CINAHL, CNKI, and the Cochrane Library, up to March 2026. Manually search for references in review articles that may meet the requirements. Two researchers independently carried out the literature search and review, data extraction and assessment of risk of bias. The analysis of results includes 6-min walk distance (6MWD), thigh circumference, knee extensor strength, gait speed, thigh muscle thickness, Chronic Liver Disease Questionnaire (CLDQ), Model for End-Stage Liver Disease (MELD) scores and handgrip strength. Statistical analyses were performed using Rev Man 5.3 and STATA 14.0.

**Results:**

This meta-analysis included 16 studies involving a total of 942 patients. The results demonstrated that exercise significantly enhanced various physical and clinical metrics in patients suffering from liver cirrhosis and sarcopenia. Notably, significant improvements were recorded in the 6MWD (MD = 54.35, 95% CI = 34.56–74.14, *P* < 0.001), thigh circumference (MD = 1.83, 95% CI = 0.92–3.38, *P* = 0.020), knee extensor strength (MD = 8.06, 95% CI = 2.59–13.53, *P* = 0.004), thigh muscle thickness (MD = 0.09, 95% CI = 0.05–0.14, *P* < 0.001), and scores on the CLDQ (MD = 0.75, 95% CI = 0.51–0.99, *P* < 0.001). Additionally, gait speed exhibited a significant increase (MD = 1.17, 95% CI = 0.09–2.26, *P* = 0.030). However, no significant changes were observed in grip strength, or MELD scores. Subgroup analysis indicates that aerobic exercise, or Resistance exercise, appear to improve physical activity levels in patients with cirrhosis.

**Conclusion:**

Exercise may improve selected indicators of muscle mass and physical performance in patients with cirrhosis. Exercise improves thigh muscle thickness, thigh circumference, and knee extension strength in patients with sarcopenia and cirrhosis, while also enhancing their physical activity capacity and quality of life. In terms of exercise type, both aerobic and resistance training appear to improve physical activity levels in patients with cirrhosis. However, further research is needed to confirm this.

**Systematic review registration:**

https://www.crd.york.ac.uk/prospero/, identifier CRD420261326379.

## 1 Introduction

Cirrhosis represents a common chronic condition linked to a significant rate of mortality. Data suggest that around 1.32 million people globally perish from this illness each year (1). Sarcopenia, a gradually advancing and prevalent disorder of skeletal muscle, is defined by a decline in muscle mass, diminished strength, and compromised physical performance. This condition can lead to a heightened likelihood of negative outcomes, such as falls, fractures, disability, and death (2, 3).

Sarcopenia frequently arises as a complication associated with cirrhosis (4, 5), and is a major contributor to morbidity and mortality among affected individuals (6–8). Research has shown that sarcopenia not only considerably heightens morbidity and mortality rates in cirrhotic patients but also adversely affects their quality of life and hampers recovery after liver transplantation (9, 10). In individuals on the waiting list for liver transplantation, the presence of sarcopenia raises the mortality risk during the waiting period, as well as the likelihood of postoperative complications and readmissions (11). Compared to patients with cirrhosis who do not have sarcopenia, those who are affected show a notably reduced overall survival rate and are at a higher risk of facing complications, such as infections (5). Therefore, finding effective ways to address the advancement of sarcopenia in cirrhosis patients has emerged as a critical area of research.

Numerous factors influence the development of sarcopenia in patients with cirrhosis. Among these, exercise, as a modifiable factor, has demonstrated potential clinical value (12). Exercise is an important non-pharmacological intervention in the management of sarcopenia for patients with cirrhosis. A meta-analysis indicated that exercise can improve physical function in these patients (13). Although no specific exercise intervention guidelines have yet been established for individuals with cirrhosis, scientifically designed exercise programs can effectively enhance physical fitness and muscle mass (14). There remains some controversy as to whether exercise is an effective intervention for sarcopenia in patients with cirrhosis. Some randomized controlled trials have shown that exercise does not significantly improve muscle mass and aerobic capacity in patients with cirrhosis (15). However, a recent meta-analysis indicated that exercise combined with protein and branched-chain amino acid (BCAA) supplementation can effectively improve muscle mass in this population (16). Nevertheless, this study did not analyze the impact of different types of exercise on sarcopenia in patients with cirrhosis.

Consequently, we conducted this meta-analysis, incorporating the most recent randomized controlled trials on exercise interventions for sarcopenia in patients with cirrhosis. The aim of this study is to further evaluate the efficacy of exercise interventions for sarcopenia in this patient population and to investigate the influence of factors such as the type of exercise through subgroup analysis.

## 2 Materials and methods

### 2.1 Protocol

This meta-analysis was conducted in accordance with the Preferred Reporting Items for Systematic Reviews and Meta-Analyses (PRISMA). Additionally, this systematic review and meta-analysis has been registered with the International Prospective Register of Systematic Reviews (PROSPERO) under registration number CRD420261326379.

### 2.2 Search strategy

A systematic literature search was conducted across five electronic databases: PubMed, Embase, CINAHL, the Cochrane Library, and CNKI, covering literature published up to March 2026. The search strategy employed a combination of Medical Subject Headings and relevant free terms, with key concepts including “Exercise” and “Liver Cirrhosis.” A detailed search strategy is provided in Supplementary Appendix.

### 2.3 Inclusion and exclusion criteria

The inclusion criteria were established according to the PICOS framework. Studies were included if they met the following conditions: (1) Population: Participants aged between 18 and 80 years who have been diagnosed with cirrhosis and who exhibit characteristics of sarcopenia as measured by at least one of the following: muscle mass, muscle strength or physical function; (2) Intervention: exercise interventions of any modality, lasting a minimum of 4 weeks; (3) Comparison: no specific intervention or standard health education; (4) Outcomes: evaluation of the 6MWD, muscle mass (thigh circumference and thigh muscle thickness), muscle strength (handgrip and knee extensor strength), gait speed, MELD scores, or CLDQ scores; and (5) Study design: randomized controlled trials.

Conversely, studies were excluded if they met any of the following criteria: (1) non-RCT designs; (2) non-original publications, such as systematic reviews, narrative reviews, commentaries, editorials, case reports, or conference abstracts; (3) missing, insufficient, or incompatible data precluding pooled analysis; or (4) animal experiments.

### 2.4 Data extraction

Two researchers independently performed the literature screening and data extraction. Any discrepancies were resolved through discussion or by consulting a third researcher. Following the initial screening of titles and abstracts, the full texts of potentially eligible studies were reviewed to confirm final inclusion. For articles with missing data, we contacted the corresponding authors via email to obtain the necessary information.

The following data were extracted from each included study: (1) Basic information: first author’s name, country of publication, and publication year; (2) Participant characteristics: sample size, age, and sex; (3) Intervention details: exercise modality (aerobic exercise or a combination of aerobic and resistance exercise), training frequency, and total duration; and (4) Outcomes: muscle mass/size (thigh circumference and quadriceps muscle thickness), muscle strength (handgrip and knee extensor strength), 6MWD, MELD scores, CLDQ scores, and gait speed. If the original data were reported as medians, ranges, or interquartile ranges (25th–75th percentiles), they were converted to means and standard deviations (SDs). When standard deviations were unavailable, we calculated them from standard errors, confidence intervals (CIs), t or *p*-values.

### 2.5 Risk of bias assessment

The methodological quality of the included studies was evaluated using the Cochrane Risk of Bias (RoB) 2.0 tool. The assessment encompassed five specific domains: bias arising from the randomization process, bias due to deviations from intended interventions, bias due to missing outcome data, bias in measurement of the outcome, and bias in selection of the reported result.

### 2.6 Certainty of evidence

The methodological quality of the evidence was systematically evaluated using the Grading of Recommendations, Assessment, Development, and Evaluations (GRADE) approach. This standardized framework allowed us to assess five domains that could potentially downgrade the quality of evidence: (1) risk of bias (study limitations), (2) inconsistency (heterogeneity), (3) indirectness of evidence, (4) imprecision, and (5) publication bias. The evidence was subsequently categorized into four quality levels: high, moderate, low, and very low.

### 2.7 Statistical methods

All statistical analyses were performed using RevMan 5.3 and Stata14.0. For continuous outcomes, the mean, standard deviation (SD), and sample size were extracted from each study. The mean difference (MD) with 95% confidence intervals (CIs) was used as the effect measure when outcomes were assessed using identical measurement units. Conversely, the standardized mean difference (SMD) was applied when different scales were utilized.

Heterogeneity among studies was evaluated using the Cochran’s Q test and the *I*^2^ statistic. A fixed-effects model was applied when heterogeneity was not significant (*P* > 0.10 and *I*^2^< 50%). If significant heterogeneity was detected (*P* < 0.10 or *I*^2^> 50%), a random-effects model was utilized.

Sensitivity analyses were performed utilizing the leave-one-out method. This approach validated the robustness of the pooled estimates and ensured that no single study disproportionately influenced the overall results. Subgroup analyses, stratified by exercise modality, total duration, and training frequency, were conducted using Stata 14.0. Publication bias was assessed visually through funnel plots. A statistical significance threshold was established at *P* < 0.05.

## 3 Results

### 3.1 Screening process

**FIGURE 1:**
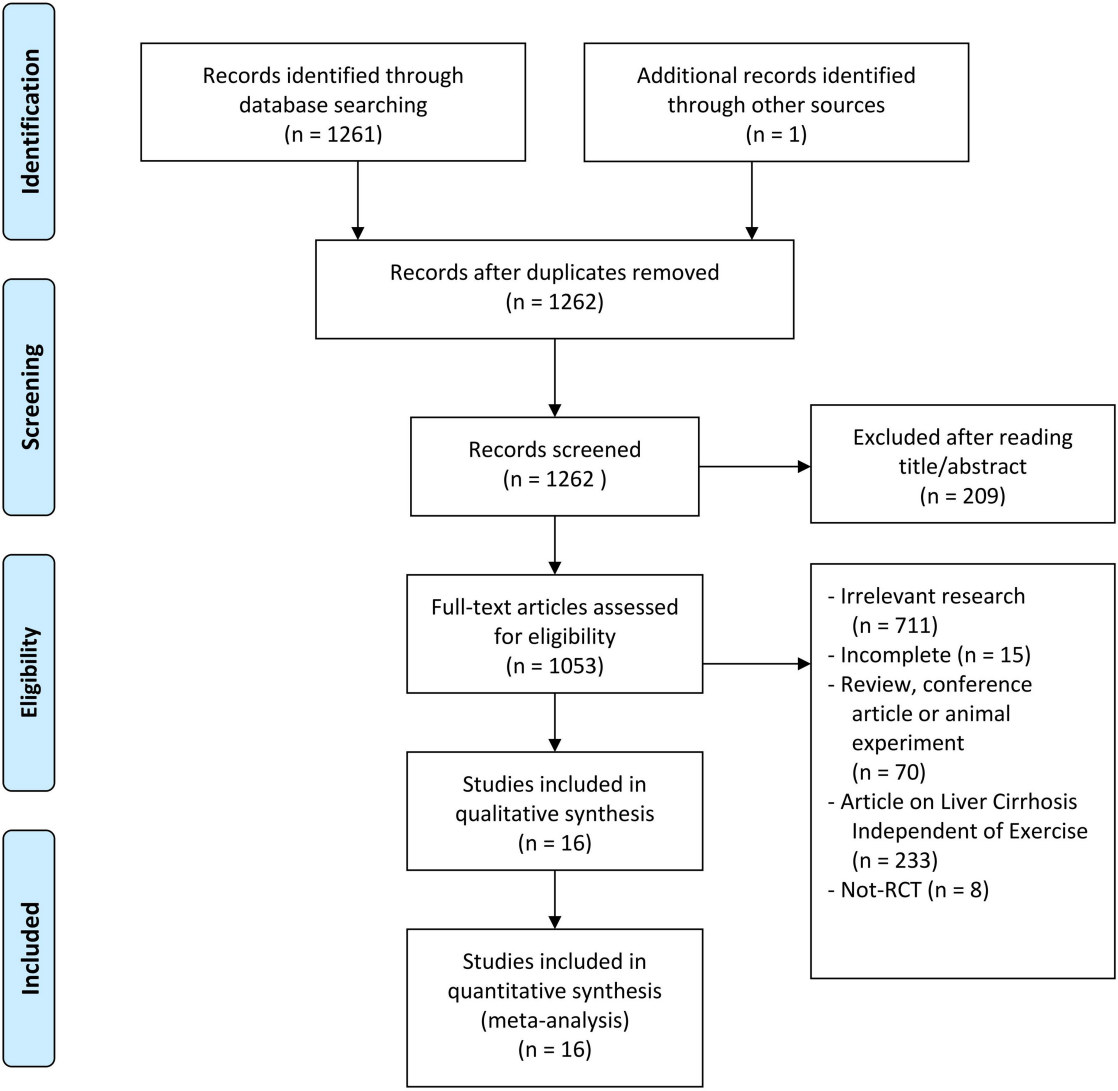
PRISMA flow diagram of RCT inclusion.

The initial literature search yielded a total of 1,262 records. After removing 209 duplicates, 1,053 articles remained for title and abstract screening. Based on this initial screening, 1,037 articles were excluded. The specific reasons for exclusion were as follows: studies unrelated to liver cirrhosis (*n* = 233), reviews, conference abstracts, or animal experiments (*n* = 70), non-RCTs (*n* = 8), other irrelevant studies (*n* = 711), and Incomplete (*n* = 15). Ultimately, 16 studies fulfilled all eligibility criteria and were included in the final meta-analysis. The literature search process is shown in Figure 1.

### 3.2 Basic characteristics of included studies

This meta-analysis included a total of 16 randomized controlled trials (Table 1) (14, 15, 17–30). Sample sizes across the eligible studies ranged from 17 to 116 patients, yielding a total of 942 enrolled participants (490 in the exercise groups and 452 in the control groups). The ages of the participants ranged from 54 to 76 years. The exercise interventions consisted of aerobic training in seven studies, resistance training in four, and a combination of both modalities in five. Intervention durations varied from 8 to 24 weeks, with training frequencies ranging from two to seven sessions per week. For the control groups, the majority of patients received standard care.

**TABLE 1:** Characteristics of the included studies.

No.	References	Country	Age (years) Exp.	Age (years) Ctrl.	N (M/F) Exp.	N (M/F) Ctrl.	Subjects	Type of exercise	Duration	Frequency	Session length	Exercise intervention	Outcome indicators
1	Zenith et al. (14)	Canada	56.4 ± 7.7	58.6 ± 5.8	9 (7/2)	10 (8/2)	Child-Pugh B or C	Aerobic exercises	8 weeks	3/week	40–60 Min	Monark cycle ergometer; power output at 60–80% of baseline peak VO2. 5-min warm-up, 30-min training (increased 2.5 min/wk).	➀➁➄➆➇
2	Chen et al. (25)	China	55.4 ± 7.6	57.6 ± 5.7	10 (7/3)	10 (8/2)	MELD > 8	Aerobic + resistance	8 weeks	3/week	60 Min	10-Min warm-up, 30-min aerobic (treadmill/cycling), 10-min resistance band training, 10-min cool-down.	➀➂➄
3	Xu et al. (30)	China	68.95 ± 5.21	70.32 ± 5.68	45 (26/19)	46 (36/14)	MELD > 7	Aerobic + resistance	16 weeks	2–3/week	20–45 Min	Aerobic warm-up, elastic band resistance and balance training. Five resistance exercises at > 60% 1RM, 2–3 sets.	➃➅
4	Jian et al. (26)	China	49.30 ± 4.66	48.12 ± 5.72	44 (31/13)	43 (29/14)	Child-Pugh A or B	Aerobic exercises	12 weeks	3/week	30 Min	Group exercises including gymnastics and slow walking; 30 min per session.	➀➂➄
5	Xie et al. (29)	China	62.05 ± 8.07	61.74 ± 7.58	41 (22/18)	40 (24/17)	Child-Pugh A/B/C	Resistance exercise	12 weeks	3/week	30–45 Min	Alternating upper/lower body resistance. 5–10 min warm-up, 20–25 min training, 5–10 min cool-down.	➃➅
6	Lin et al. (27)	China	55.68 ± 7.00	57.11 ± 8.75	28 (18/10)	27 (16/11)	Child-Pugh A or B	Aerobic exercises	12 weeks	4–5/week	30–40 Min	Brisk walking, ballroom dancing, table tennis and other recreational activities.	➀
7	Román et al. (23)	Spain	65.5	61	8 (5/3)	9 (7/2)	Child-Pugh A or B	Aerobic exercises	12 weeks	3/week	60 Min	Treadmill or stationary cycling. 60 min per session including 10-min warm-up.	➀➁
8	Wang et al. (28)	China	69.55 ± 5.98	69.38 ± 5.21	47 (25/22)	47 (29/18)	Cirrhosis	Aerobic exercises	24 weeks	7/week	30–60 Min	Slow/fast walking, cycling, jogging, or rope skipping. 15-min preparation, 30–60 min exercise, 10-min cool-down.	➃➅
9	Chen et al. (19)	United States	55 ± 7	54 ± 11	9 (5/4)	8 (6/2)	Child-Pugh B or C	Aerobic exercises	12 weeks	7/week	30 Min	Walking-based home exercise. Baseline daily steps + 500 steps/day, increased weekly or biweekly.	➀
10	Kruger et al. (20)	Canada	53.0 ± 8.3	56.4 ± 8.5	20 (10/10)	20 (13/7)	Child-Pugh A or B	Aerobic exercises	8 weeks	3/week	30–60 Min	Stationary cycling exercise (Schwinn stationary bicycle).	➀➁➆➇
11	Aamann et al. (17)	Denmark	61.7 ± 7.8	63 ± 7.0	19 (16/4)	15 (14/5)	Child-Pugh A or B	Resistance exercise	12 weeks	3/week	60 Min	5-Min warm-up + 7 full-body resistance exercises.	➀➁➂➇
12	Sirisunhirun et al. (15)	Thailand	55.6 ± 8.9	57.1 ± 6.7	20 (15/5)	20 (11/9)	Child-Pugh B or C	Aerobic + resistance	14 weeks	4/week	40 Min	5-Min warm-up, 30-min aerobic and isotonic exercise, 5-min cool-down.	➀➁➆
13	Sobhy et al. (24)	Egypt	62.18 ± 8.03	59.80 ± 8.04	55 (35/20)	55 (35/20)	Child-Pugh B or C	Aerobic + resistance	4 weeks	Aerobic 4–7/week; resistance 2/week	30 Min	Aerobic: Walk 1 min, rest 1 min, gradually increase to 20 min/day. Upper/lower/trunk resistance training.	➅➇
14	Brown et al. (18)	Brazil	58.9 ± 10.1	59.6 ± 8.8	19 (12/7)	19 (12/7)	Child-Pugh A or B	Resistance exercise	12 weeks	2/week	40 Min	Resistance training: 40-min session, 1-min rest between sets, 5-min stretching cool-down.	➀➅
15	Lai et al. (3)	United States	62 (56–66)	61 (42–72)	58 (29/29)	25 (18/7)	Child-Pugh A/B/C	Aerobic + resistance	12 weeks	3/week	30 Min	Three weekly resistance training sessions and 500 additional steps/week over the preceding week.	➆
16	Ma et al. (22)	China	63.43 ± 3.12	64.26 ± 2.57	58 (30/28)	58 (27/31)	Child-Pugh A or B	Aerobic + resistance	12 weeks	3/week	65 Min	Warm-up, resistance training, aerobic exercise (brisk walking, jogging, cycling, dancing) and cool-down.	➃➅

Exp., Exercise group; Ctrl., Control group; M/F, Male or Female; ➀6-min walk distance; ➁Thigh circumference; ➂Knee extensor strength; ➃Gait speed; ➄Thigh muscle thickness; ➅Handgrip strength; ➆Chronic Liver Disease Questionnaire; ➇Model for End-Stage Liver Disease; Among these, ➀➁➂➃➄➅are primary outcome measures, and ➆➇are secondary outcome measures.

### 3.3 Risk of bias

**FIGURE 2:**
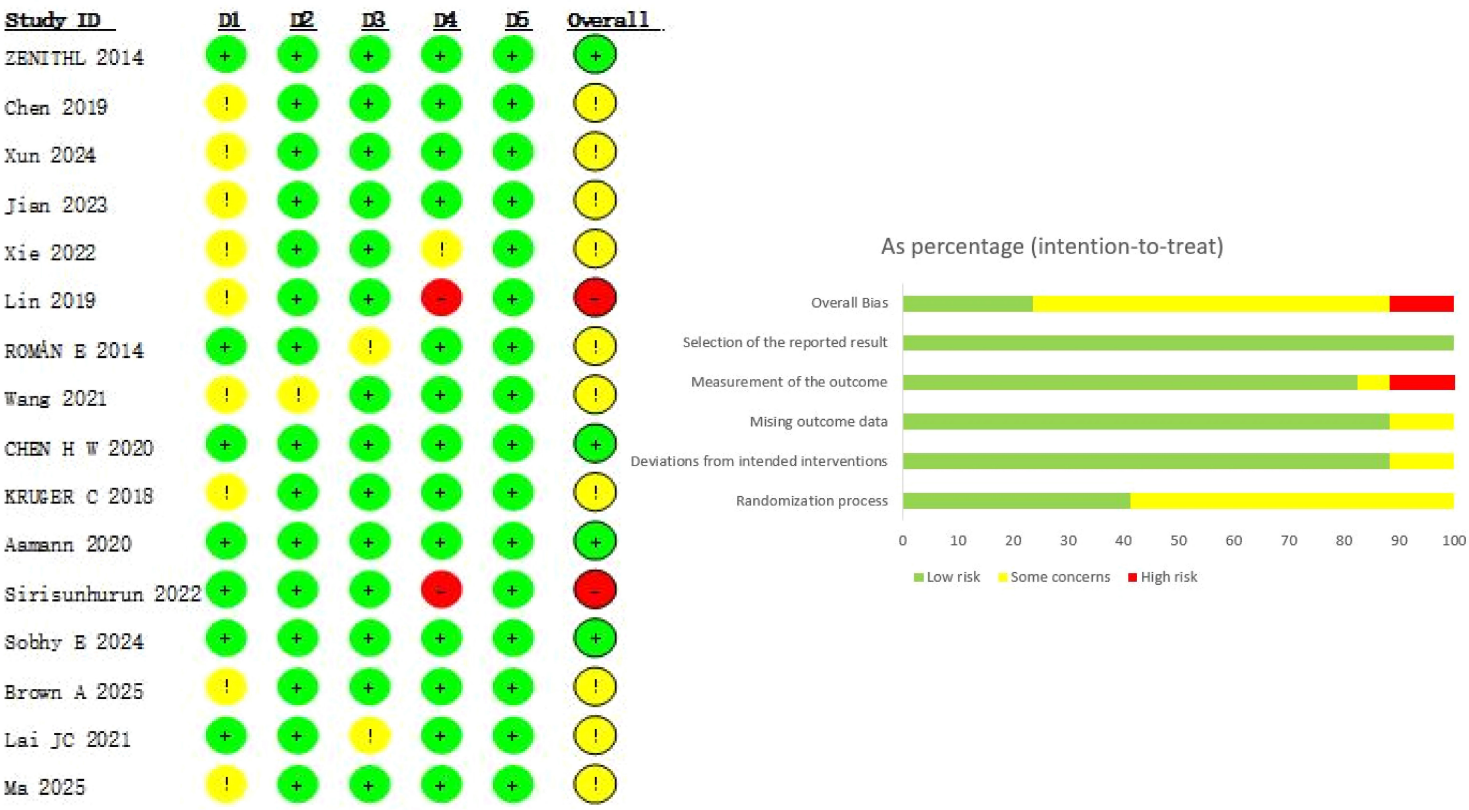
Risk of bias summary.

A bias analysis of the included studies revealed that 10 studies (62.5%) had a moderate risk of bias, 4 studies (25%) had a low risk of bias, and 2 studies (12.5%) had a high risk of bias. The main factors influencing this risk assessment were potential bias in allocation concealment and outcome measurement. In studies involving exercise interventions, it is difficult for participants and researchers to implement complete blinding, which affects the accuracy of the results (Figure 2).

### 3.4 6 WMD

**FIGURE 3:**
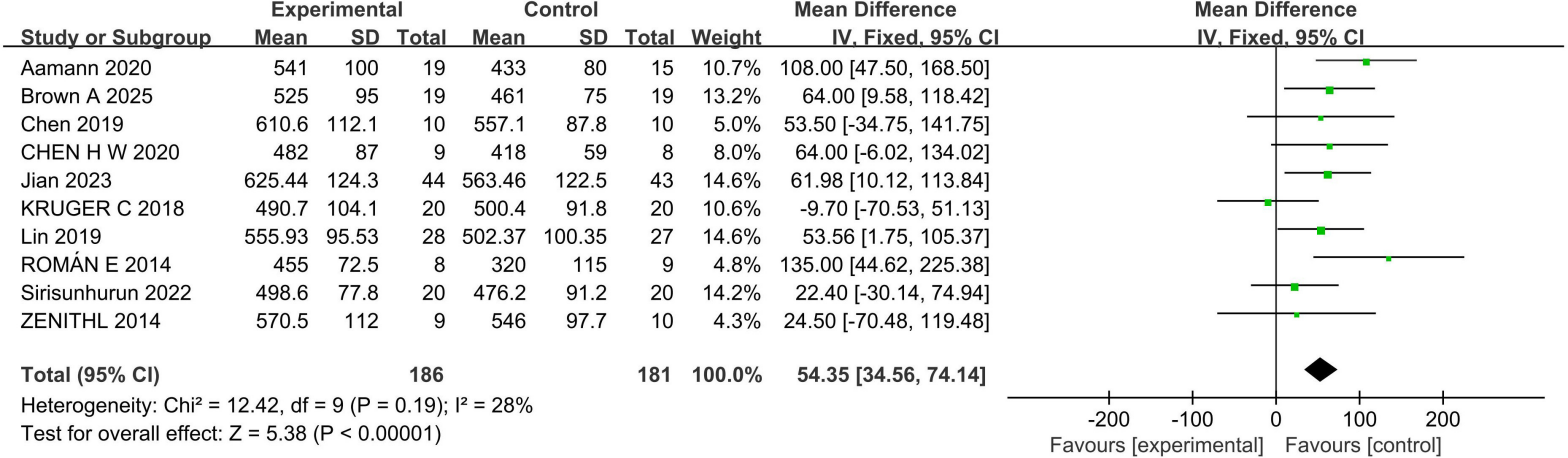
Forest plot of 6WMD.

Ten studies reported data on the 6WMD. The pooled results indicated that exercise interventions significantly increased the 6WMD in patients with liver cirrhosis and sarcopenia (MD = 54.35, 95% CI = 34.56**–**74.14, *p* < 0.001, *I*^2^ = 28%, Figure 3). Sensitivity analysis results demonstrated that, after sequentially excluding studies, the range of the MD varied from 47.92 to 61.93, suggesting the stability of the findings (Supplementary Figures 6, 7).

### 3.5 Thigh circumference

**FIGURE 4:**
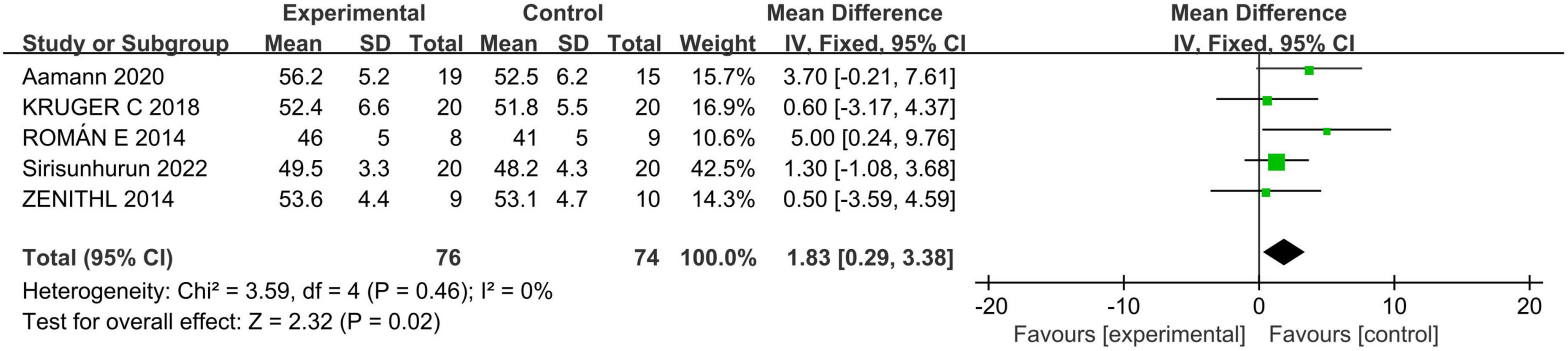
Forest plot of thigh circumference.

Five studies reported data on thigh circumference. The pooled analysis revealed a significant increase in thigh circumference following exercise interventions in patients with liver cirrhosis and sarcopenia (MD = 1.83, 95% CI = 0.92–3.38, *p* = 0.020, *I*^2^ = 0%, Figure 4). Sensitivity analysis confirmed the stability of this outcome. Following the leave-one-out procedure, the pooled MD ranged from 1.46 to 2.23 (Supplementary Figures 8, 9).

### 3.6 Knee extensor strength

**FIGURE 5:**
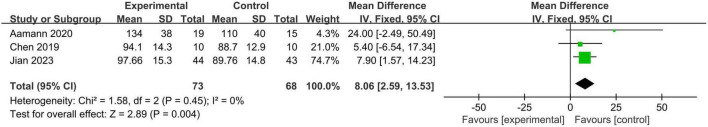
Forest plot of knee extensor strength.

Three studies reported data on knee extensor strength. Pooled analysis revealed a significant increase in knee extensor strength following exercise interventions in patients with liver cirrhosis and sarcopenia (MD = 8.06, 95% CI = 2.59**–**13.53, *p* = 0.004, *I*^2^ = 0%, Figure 5). Sensitivity analysis confirmed the stability of this outcome. Following the leave-one-out procedure, the pooled MD ranged from 7.35 to 8.77 (Supplementary Figures 10, 11).

### 3.7 Gait speed

**FIGURE 6:**
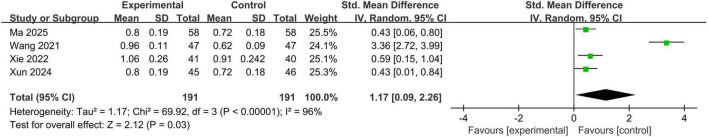
Forest plot of gait speed.

Four studies reported data on gait speed. Given the substantial heterogeneity among these trials (*I*^2^ = 96%), a random-effects model was applied. Pooled analysis demonstrated a significant increase in gait speed following exercise interventions in patients with liver cirrhosis and sarcopenia (MD = 1.17, 95% CI = 0.09**–**2.26, *p* = 0.030, *I*^2^ = 96%, Figure 6). Sensitivity analysis indicated that this outcome remained robust. Following the leave-one-out procedure, the pooled MD ranged from 0.47 to 1.44 (Supplementary Figures 12, 13).

### 3.8 Thigh muscle thickness

**FIGURE 7:**
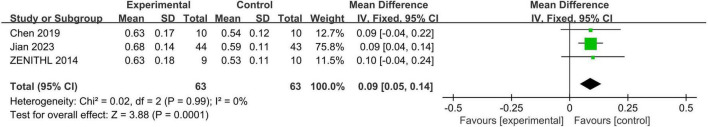
Forest plot of thigh muscle thickness.

Three studies reported data on thigh muscle thickness. Pooled analysis demonstrated a significant increase in thigh muscle thickness following exercise interventions in patients with liver cirrhosis and sarcopenia (MD = 0.09, 95% CI = 0.05–0.14, *p* < 0.001, *I*^2^ = 0%, Figure 7). Sensitivity analysis confirmed the stability of this outcome. Following the leave-one-out procedure, the pooled MD remained consistent at 0.09 (Supplementary Figures 14, 16).

### 3.9 Handgrip strength

**FIGURE 8:**
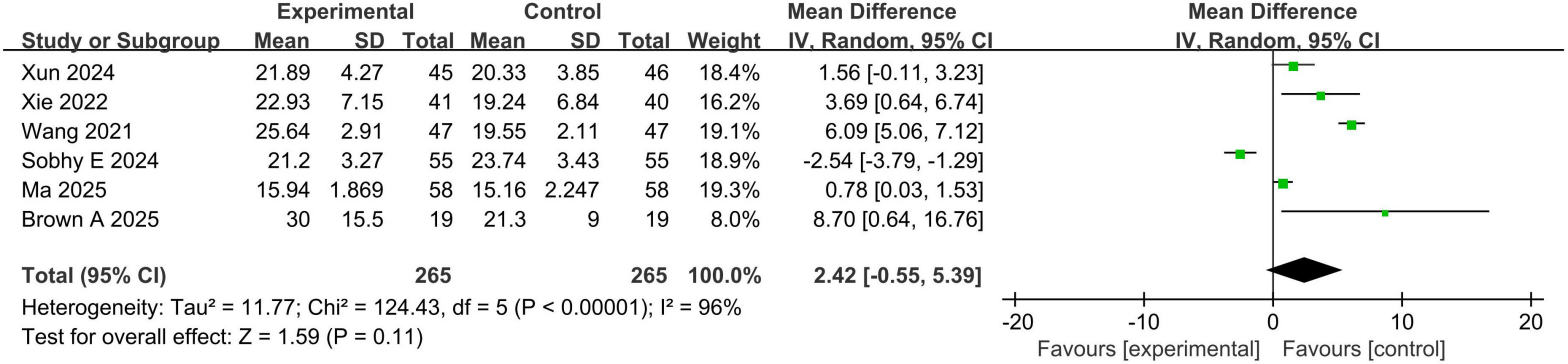
Forest plot of hand grip strength.

Six studies reported data on handgrip strength. Given the substantial heterogeneity among these trials (*I*^2^ = 96%), a random-effects model was applied. Pooled analysis indicated that exercise interventions had no significant effect on handgrip strength in patients with liver cirrhosis and sarcopenia (MD = 2.42, 95% CI = **−**0.55 to 5.39, *p* = 0.110, *I*^2^ = 96%, Figure 8). Following the leave-one-out procedure for sensitivity analysis, the pooled MD ranged from 1.12 to 3.51 (Supplementary Figures 17, 18).

### 3.10 Subgroup analysis

Subgroup analysis was conducted by categorizing the ten studies into three exercise modalities: aerobic exercise, resistance exercise, and a combination of aerobic and resistance exercise (Supplementary Figures 23). The pooled results from two studies indicated that the resistance exercise significantly improved the 6WMD (MD = 83.68, 95%, CI = 43.22–124.14, *p* < 0.001, *I*^2^ = 10.9%). Similarly, aerobic exercise, as reported in six studies, significantly enhanced the 6MWD (MD = 50.56, 95% CI = 23.81–76.30, *p* < 0.001, *I*^2^ = 35.5%). In contrast, combination of aerobic and resistance exercise, evaluated in two studies, did not demonstrate a significant effect on the 6MWD (MD = 30.54, 95% CI = −14.61–75.68, *p* = 0.185, *I*^2^ = 0%).

The 10 studies were divided into two groups based on the total duration of intervention: ≤ 8 weeks and > 8 weeks (Supplementary Figure 24). The results of the meta-analysis of 7 studies showed that training lasting ≤ 8 weeks significantly improved the 6MWD in patients with sarcopenia due to cirrhosis (MD = 72.20, 95% CI = 19.61–124.78, *p* = 0.007, *I*^2^ = 33.2%). The pooled results of 3 studies showed that training lasting more than 8 weeks significantly improved the 6MWD in patients with sarcopenia due to cirrhosis (MD = 51.40, 95% CI = 30.04–72.76, *p* < 0.001, *I*^2^ = 32.6%).

The 10 studies were divided into two groups based on intervention frequency: those with 3 or fewer sessions per week and those with more than 3 sessions per week (Supplementary Figure 25). Seven studies reported interventions of three times or fewer per week. The pooled results indicated that training three times or fewer per week significantly improved the 6MWD in patients with sarcopenia due to cirrhosis, although there was heterogeneity within the group (MD = 60.48, 95% CI = 35.59–85.37, *p* < 0.001, *I*^2^ = 43.9%). Three studies reported on interventions involving more than 3 sessions per week. The pooled results indicated that exercise significantly improved the 6MWD in patients with sarcopenia and cirrhosis (MD = 43.80, 95% CI = 11.16–76.44, *p* = 0.009, *I*^2^ = 0%).

### 3.11 Publication bias

A funnel plot was used to assess publication bias (Supplementary Figure 1–5). Analysis revealed no significant publication bias in the studies examining 6MWD, thigh circumference, knee extensor strength, CLDQ scores and thigh muscle thickness.

### 3.12 Certainty of evidence

According to the GRADE framework, the quality of evidence was rated as moderate for the 6MWD, thigh circumference, and CLDQ scores, with these outcomes being downgraded primarily due to the risk of bias. Conversely, the evidence was rated as low quality for thigh muscle thickness and knee extensor strength (downgraded for both risk of bias and imprecision), as well as for gait speed, MELD scores, and handgrip strength (downgraded for risk of bias and inconsistency) (Supplementary Table 3).

## 4 Discussion

The results of this study demonstrate that exercise significantly enhances the 6-min walk test, thigh circumference, thigh muscle thickness, knee extension strength, and walking speed in patients with cirrhosis. Additionally, exercise effectively improves the quality of life for these patients (Supplementary Figure 27). However, when evaluating the effects of the exercise intervention through grip strength and MELD scores (Supplementary Figures 28), no statistically significant differences were observed in the corresponding outcomes. Subgroup analyses further indicated that aerobic exercise or resistance exercise appear to influence physical performance in patients with cirrhosis. However, given the limited number of studies in each subgroup, these findings should be considered preliminary and exploratory. Due to the lack of clear patient data, we are unable to reliably perform additional subgroup analyses based on the Child-Pugh classification. This has also been identified as an important area for future research. The differences between aerobic exercise, resistance training, and combined exercise regimens still need to be validated through larger-scale randomized trials.

6WMD is a recognized prognostic indicator for patients with cirrhosis, and its results are closely associated with overall mortality. Enhancing lower limb motor function can effectively reduce the risks faced by this vulnerable population during walking (31, 32). This study found that patients with sarcopenia and cirrhosis showed a significant improvement in their 6WMD following exercise intervention. A previous study reached a similar conclusion. The study indicates that resistance training combined with aerobic exercise can reduce the risk of serious adverse events in patients with cirrhosis. The results of related studies suggest that exercise therapy can be safely implemented and may help improve the prognosis of patients with cirrhosis. Taken together, these findings support the role of exercise as an important non-pharmacological strategy for improving outcomes in cirrhosis (33). However, some studies have contested these findings, with results indicating that exercise intervention alone has no significant effect on patients’ 6WMD (34, 35). This discrepancy may be attributed to considerable variations in the intensity, methodology and duration of the exercise programs used in these studies. Subgroup analyses indicated that resistance exercise or aerobic exercise effectively improved the 6WMD. Studies have shown that resistance exercise can improve sarcopenia in patients with cirrhosis (36). A previous review suggested that the combined use of exercise and amino acid supplementation can enhance patients’ 6WMD, although it may also carry a risk of elevated blood glucose levels (37). Subgroup analysis results demonstrated that different frequencies and durations of exercise can all effectively improve physical function in patients with cirrhosis.

Research has found that exercise can improve thigh circumference and thigh muscle thickness in patients with cirrhosis. Previous studies have similarly demonstrated that exercise is effective in improving key indicators such as thigh circumference and quadriceps thickness in patients with cirrhosis (34, 37, 38). Four RCTs in this study demonstrated that interventions incorporating resistance exercise can effectively improve thigh circumference in patients (14, 15, 17, 20). Resistance exercise promotes skeletal muscle hypertrophy by causing muscles to contract against external resistance (39). At the same time, resistance training can also stimulate muscle protein synthesis, thereby increasing muscle mass (40). A previous review indicated that exercise positively effects on muscle mass in patients with cirrhosis (41). An early trial reported that exercising three times a week for 1 h per session significantly improved muscle mass and physical performance in patients with cirrhosis, while also increasing their lean body mass (42). An increase in lean body mass contributes to improved functional capacity of skeletal muscle mitochondria, thereby generating greater energy (14). Consequently, modulating structural muscle hypertrophy through exercise forms the foundation for maintaining strength and enhancing health status.

Reduced muscle strength and functional limitations are the primary factors contributing to an increased risk of falls in patients with cirrhosis (43). Knee extensor strength, recognized as the gold standard for assessing muscle strength in chronic liver disease (44), has demonstrated superior predictive value for functional performance in older adults (45). Studies have shown that exercise can improve knee extensor strength in these patients. However, it is important to note that the sample sizes were small, and the results should be interpreted with caution. Knee extensor strength is associated with balance and overall strength in older adults (46). By enhancing knee extensor strength and restoring local muscle support capacity (33), patients’ balance can be improved, effectively reducing the risk of falls.

This study found that exercise can significantly enhance the quality of life for patients with cirrhosis. Patients with cirrhosis often experience symptoms such as muscle cramps, which adversely affect their quality of life (47). Additionally, impaired physical function further diminishes these patients’ quality of life (48). Exercise not only alleviates fatigue in patients with cirrhosis but also improves their overall quality of life by addressing issues of frailty (49). Beyond enhancing physical capacity, physical activity stimulates the release of endogenous endorphins (50). The analgesic properties of these neurotransmitters contribute to mood stabilization, thus positively influencing patients’ quality of life.

Possibly due to methodological limitations, no statistically significant differences were found in the results for MELD score and grip strength. Furthermore, the significant heterogeneity observed in the results for walking speed, grip strength and MELD score makes it difficult to draw definitive conclusions regarding these outcomes. The CT-based skeletal muscle index (SMI) is considered one of the most objective and validated measures for assessing sarcopenia in patients with cirrhosis. However, only a few of the included studies reported CT-based assessments of muscle mass. Although grip strength plays an important role in the AWGS and JSH criteria, no significant improvement was observed, suggesting that there is currently insufficient evidence to conclude that exercise universally improves sarcopenia. At the same time, the high heterogeneity in gait speed also limits the interpretability of the meta-analysis. Therefore, the results of the current study should be interpreted with caution.

The results of this meta-analysis have several limitations. Firstly, there were variations in the exercise programs across the included studies, making it difficult to establish uniform standards for the intensity, duration and frequency of the interventions. Secondly, the severity of cirrhosis varied among patients, limiting the generality of the study’s findings. Thirdly, publication bias is inevitable during the literature search and selection process. Fourthly, as SMI is regarded as a reference standard for sarcopenia assessment in cirrhosis, the lack of sufficient SMI-based outcomes restricted our ability to evaluate the effect of exercise on objectively measured skeletal muscle mass. Finally, the insufficient number of data points included in the analysis of certain outcomes has imposed certain limitations. Taking these factors into account, future studies should incorporate more high-quality randomized controlled trials to improve the quality of the results.

## 5 Conclusion

Exercise improves thigh muscle thickness, thigh circumference, and knee extension strength in patients with sarcopenia and cirrhosis, while also enhancing their physical activity capacity and quality of life. In terms of exercise types, aerobic exercise or resistance exercise appear to improve physical activity levels in patients with cirrhosis.

## Data Availability

The original contributions presented in this study are included in this article/Supplementary material, further inquiries can be directed to the corresponding authors.
