# Supplementary material for: The impact of exercise on improving sarcopenia in patients with liver cirrhosis: a systematic review and meta-analysis
Source: Front Med (Lausanne). 2026 Jul 29;13:1871179. doi: 10.3389/fmed.2026.1871179 (PMC13461329; doi:10.3389/fmed.2026.1871179)
Supplement: Supplementary file 1 [file Data_Sheet_1.docx]

**Appendix**

*********Evidence from meta-analysis**

**S1. Search strategy**

**Table S1 Search terms (appropriate adjustment for each database)**

| **Number** | **Terms** |
| --- | --- |
| #1 | Liver Cirrhosis[Mesh] OR (Cirrhosis, Liver):ti,ab,kw OR (Hepatic Cirrhosis):ti,ab,kw OR (Cirrhosis, Hepatic):ti,ab,kw OR (Liver Fibrosis):ti,ab,kw OR (liver cirrhosis):ti,ab,kw OR (Cirrhosis):ti,ab,kw OR (Liver Cirrhosis):ti,ab,kw |
| #2 | Exercise[Mesh] OR (Physical Exercise):ti,ab,kw OR (Training):ti,ab,kw OR (Exercise Training):ti,ab,kw OR (Physical Activity):ti,ab,kw OR (Exercises):ti,ab,kw OR (Isometric Exercise):ti,ab,kw OR (Aerobic Exercise):ti,ab,kw |
| #3 | "randomized-controlled trials":ti,ab,kw OR ("randomized"):ti,ab,kw OR (placebo):ti,ab,kw |
| #4 | #1 AND #2 AND #3 |

**S2. Funnel plots**

**Fig S1 Funnel plot of the effect of exercise on 6-minute walk distance (6MWD)**

**Fig S2 Funnel plot of the effect of exercise on thigh circumference**

**Fig S3 Funnel plot of the effect of exercise on knee extension muscle strength**

**Fig S4 Funnel plot of the effect of exercise on CLDQ**

**Fig S5 Funnel plot of the effect of exercise on muscle thickness**

**S3. Leave-one-out sensitivity analysis**

**Fig S6 The forest plot of leave-one-out analysis for 6MWD (maximum effect)**

**Fig S7 The forest plot of leave-one-out analysis for 6MWD (minimum effect)**

**Fig S8 The forest plot of leave-one-out analysis for thigh circumference (maximum effect)**

**Fig S9 The forest plot of leave-one-out analysis for thigh circumference (minimum effect)**

**Fig S10 The forest plot of leave-one-out analysis for knee extension muscle strength (maximum effect)**

**Fig S11 The forest plot of leave-one-out analysis for knee extension muscle strength (minimum effect)**

**Fig S12 The forest plot of leave-one-out analysis for gait speed (maximum effect)**

**Fig S13 The forest plot of leave-one-out analysis for gait speed (minimum effect)**

**Fig S14 The forest plot of leave-one-out analysis for thigh muscle thickness (1)**

**Fig S15 The forest plot of leave-one-out analysis for thigh muscle thickness (2)**

**Fig S16 The forest plot of leave-one-out analysis for thigh muscle thickness (3)**

**Fig S17 The forest plot of leave-one-out analysis for handgrip strength (maximum effect)**

**Fig S18 The forest plot of leave-one-out analysis for handgrip strength (minimum effect)**

**Fig S19 The forest plot of leave-one-out analysis for CLDQ (maximum effect)**

**Fig S20 The forest plot of leave-one-out analysis for CLDQ (minimum effect)**

**Fig S21 The forest plot of leave-one-out analysis for MELD (maximum effect)**

**Fig S22 The forest plot of leave-one-out analysis for MELD (minimum effect)**

**S4. Meta-regression results**

**Table S2 Meta-regression results**

| **Variable** | **exp(b)** | **Std. Err.** | **t** | **P>\|t\|** | **[95% Conf. Interval]** |
| --- | --- | --- | --- | --- | --- |
| Exercise mode | 1.91e-12 | 3.19e-11 | -1.61 | 0.145 | 3.44e-29 to 105958.9 |
| _cons | 3.26e+46 | 1.12e+48 | 3.11 | 0.014 | 1.05e+12 to 1.01e+81 |
| Duration (weeks) | 1.32e-09 | 4.42e-08 | -0.61 | 0.558 | 4.04e-43 to 4.33e+24 |
| _cons | 1.43e+40 | 8.94e+41 | 1.48 | 0.177 | 4.35e-23 to 4.7e+102 |
| Frequency (times/week) | 8.90e-08 | 2.37e-06 | -0.61 | 0.559 | 1.91e-34 to 4.16e+19 |
| _cons | 3.11e+33 | 1.19e+35 | 2.02 | 0.078 | .0000196 to 4.94e+71 |

**S5. Subgroup analysis**

**Fig S23 Forest plot of subgroup analysis by exercise type**

Subgroup analysis was performed by exercise mode (aerobic training, resistance training, and combined aerobic and resistance training). The results showed that combined aerobic and resistance training could improve sarcopenia in patients with liver cirrhosis (MD=83.68, 95%CI= 43.22 to 124.14, p<0.001, I²=10.9%). Aerobic training could improve sarcopenia (MD=50.56, 95%CI= 23.81 to 76.30, p<0.001, I²=35.5%). Resistance training alone showed a non-significant effect (MD=30.54, 95%CI= -14.61 to 75.68, p=0.185, I²=0%).

**Fig S24 Forest plot of subgroup analysis by duration**

Subgroup analysis was performed by duration (<=8 weeks and >8 weeks). The results showed that both short-term (<=8 weeks, MD=72.20, 95%CI= 19.61 to 124.78, p=0.007, I²=33.2%) and long-term (>8 weeks, MD=51.40, 95%CI=30.04 to 72.76, p<0.001, I²=32.6%) .

**Fig S25 Forest plot of subgroup analysis by exercise frequency**

Subgroup analysis was performed by exercise frequency (<=3 times/week and >3 times/week). The results showed that both lower frequency (<=3 times/week, MD=60.48, 95%CI= 35.59 to 85.37, p<0.001, I²=43.9%) and higher frequency (>3 times/week, MD=43.80, 95%CI= 11.16 to 76.44, p=0.009, I²=0%).

**S6. The results of GRADE assessment**

**Table S3 GRADE assessment**

| **Outcomes** | **Risk of Bias** | **Inconsistency** | **Indirectness** | **Imprecision** | **Other Considerations** | **Quality** |
| --- | --- | --- | --- | --- | --- | --- |
| 6WMD | Serious | No | No | No | No | Moderate |
| Muscle thickness | Serious | No | No | Serious | No | Low |
| Thigh circumference | Serious | No | No | No | No | Moderate |
| Knee extension muscle strength | Serious | No | No | Serious | No | Low |
| Leg speed | Serious | Serious | No | No | No | Low |
| Grip | Serious | Serious | No | No | No | Low |
| BMI | Serious | No | No | Serious | No | Low |
| CLDQ | Serious | No | No | No | No | Moderate |
| MELD | Serious | Serious | No | No | No | Low |

**S7. Secondary outcomes**

**Fig S26 CLDQ**

Four studies reported data on CLDQ scores. Pooled analysis demonstrated a significant improvement in CLDQ scores following exercise interventions in patients with liver cirrhosis and sarcopenia (MD=0.75, 95%CI= 0.51 to 0.99, p<0.001, I^2^=47%). Sensitivity analysis confirmed the stability of this outcome. Following the leave-one-out procedure, the pooled MD ranged from 0.54 to 0.82 (Appendix, S3, Figs. S19-20).

**Fig S27 MELD**

Four studies reported data on MELD scores. Given the significant heterogeneity among these trials (I2=54%), a random-effects model was applied. Pooled analysis indicated that exercise interventions had no significant effect on MELD scores in patients with liver cirrhosis and sarcopenia (MD=0.16, 95%CI=-1.22 to 1.54, p=0.82, I^2^=54%). Following the leave-one-out procedure for sensitivity analysis, the pooled MD ranged from -0.16 to 0.62 (Appendix, S3, Figs. S21-22).
